# Feasibility of Bioimpedance Analysis to Assess the Outcome of Complex Decongestive Therapy in Cancer Treatment-Related Lymphedema

**DOI:** 10.3389/fonc.2020.00111

**Published:** 2020-02-11

**Authors:** Kye Hee Cho, Eun Young Han, Seung Ah Lee, Hyun Park, Chan Lee, Sang Hee IM

**Affiliations:** ^1^Department of Rehabilitation Medicine, CHA Gumi Medical Center, CHA University, Gumi, South Korea; ^2^Department of Rehabilitation Medicine, School of Medicine, Jeju National University, Jeju, South Korea; ^3^Department of Surgery, CHA Bundang Medical Center, CHA University, Seongnam, South Korea; ^4^Department of Obstetrics and Gynecology, CHA Bundang Medical Center, Seongnam, South Korea; ^5^Department and Research Institute of Rehabilitation Medicine, Severance Hospital, Yonsei University College of Medicine, Seoul, South Korea

**Keywords:** cancer treatment-related lymphedema, bioimpedance analysis, complex decongestive therapy, breast cancer, gynecologic cancer

## Abstract

**Background:** Cancer treatment-related lymphedema (CTRL) affects patients physically, psychologically and emotionally, and remains a significant quality of life issue among patients with cancer. Reliable methods to measure changes in lymphedema are required for early detection, acute intensive treatment, and long-term management. Here, we evaluated the use of bioimpedance analysis (BIA) as a tool to measure lymphedema before and after treatment.

**Patients and Methods:** Patients with CTRL who were admitted to a secondary university hospital between October 2017 and July 2018 for complex decongestive therapy (CDT) were eligible for this prospective cohort study. Circumferential measure (CM) and BIA were used to evaluate lymphedema at admission (initial) and before discharge (follow-up, FU). Volume was calculated from the CM using the truncated cone formula. The inter-limb ratios (ILRs) of the circumference, volume, and impedance were also calculated as the unaffected limb to affected limb. Each parameter before and after treatment and correlations between parameters also were analyzed.

**Results:** A total of 29 patients (12 upper- and 17 lower-extremity CTRL) completed were included in this analysis. Absolute value and the ILRs of circumference, volume or impedance, and extracellular water/total body water (ECW/TBW) were significantly improved at FU (*p* < 0.01, *p* < 0.05). The initial and FU absolute values, ILRs, ECW/TBW correlated significantly with each other (*p* < 0.01, *p* < 0.05). The cutoff values of ECW/TBW for moderate and severe degree of CTRL were 0.3855 and 0.3955, respectively. The changes of ILRs between initial and FU assessments were significantly different among three groups according to lymphedema severity (*p* < 0.01, *p* < 0.05).

**Conclusions:** BIA data correlates significantly with clinical measurement, and therefore can be a practical tool in monitoring outcome measure after lymphedema treatment. In addition, BIA is more sensitive to subtle changes in lymphedema, and therefore can be useful for the long-term maintenance of lymphedema.

## Introduction

As survival among patients with breast and gynecological cancers has improved over the past few decades (1–3), greater emphasis has been directed toward long-term cancer treatment sequelae, such as lymphedema. Cancer treatment-related lymphedema (CTRL) develops due to the disturbance of lymphatic flow by surgery and/or radiation therapy. Although the reported incidence varies across studies, 13–42% of patients with breast cancer and 20–50% of patients with gynecologic cancer are reported to suffer from lymphedema (4–8). The development of lymphedema results in physical impairments including compromised function, diminished strength, fatigue, and pain in the affected limb as well as negative psychological and emotional effects, including anxiety, frustration, sadness, anger, fear, and decreased self-confidence. Thus, due to its significant impact on quality of life (9–11), early detection and intensive treatment of CTRL is needed.

Several methods to evaluate lymphedema are currently used. A measuring tape is used for circumferential measurement (CM) of the affected limb (which in turn is used to calculate the volume via the truncated cone formula). The CM is widely used to evaluate lymphedema because it is cost-effective and easy to implement (12, 13). There are major concerns with CM, however, including the variance in inter-rater and intra-rater reliability and the difficulty in detecting early lymphedema changes. Imaging methods, such as lymphoscintigraphy and magnetic resonance (MR) lymphangiography, have several drawbacks, including pain, cost, and time. Ultrasonography also has several disadvantages: it can detect structural but not physiological changes, it is costly, there is no standardized evaluation method, and the site of evaluation is difficult to determine.

Bioimpedance analysis (BIA) has been introduced as a new method for evaluating lymphedema in the early 1990's (14). BIA is easy to implement and enables indirect quantification of extracellular fluid via the response of the body to an applied electrical current. The examiner does not need special skills to conduct BIA, and the test can be conducted in 3–5 min. Several studies have reported that BIA is correlated with measurements from CM and ultrasonography, and that BIA can be used for early diagnosis of lymphedema (15–18). Here, we extend the findings of these prior studies to validate the use of BIA in the assessment of lymphedema after phase one of complex decongestive treatment (CDT). In contrast to previous studies, in which the test results were compared at one time point, here we conduct serial comparisons to determine the utility of BIA in evaluating lymphedema longitudinally, including as a reliable measure of treatment outcome.

## Methods

### Participants

This was a prospective cohort study of 54 patients with CTRL who were admitted to the secondary university hospital for short-term (i.e., 2–6 weeks) treatment of lymphedema between October 2017 and July 2018.

Lymphedema was diagnosed by clinical and lymphoscintigraphic evaluation. Lymphedema was confirmed when the circumference of the affected limb exhibited a 5% change in volume or was <2 cm at any site compared with the unaffected limb at two consecutive assessments. Lymphedema was diagnosed if any subjective symptoms and abnormalities on the lymphoscintigraphy were present at the same time, regardless of the circumference or volume criteria. The patient's subjective symptoms, including swelling, heaviness, fullness, and vague pain were considered in the clinical diagnosis. A diagnosis via lymphoscintigraphy included decreased clearance of injection site, asymmetric visualization of lymphatic or collateral vessels, presence of dermal back flow, and reduced or no uptake of radiotracer on lymph nodes.

Among eligible patients, only those who agreed to participate in the study and who underwent CDT were included. Patients were excluded for: (1) bilateral lymphedema, (2) history of orthopedic surgery on the affected limb prior to the development of lymphedema, (3) recurrent cancer, (4) vascular disease, and (5) systemic disease associated with limb edema.

**Figure 1:**
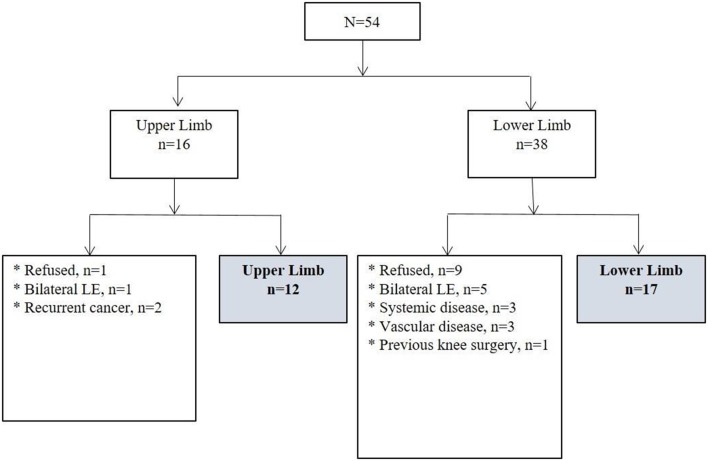
Diagram of participants. LE, lymphedema.

A total of 29 patients with unilateral lymphedema (12 upper-extremity and 17 lower-extremity) were included in the analysis. Of those excluded from the analysis, 6 patients had bilateral lymphedema; 3 had a combination of exclusionary medical conditions, such as chronic kidney disease, heart disease, and stroke; 3 had vascular disease; 2 had recurrent cancer; and 1 had a previous knee surgery on the affected limb. Ten patients who did not agree to participate in the study also were excluded (Figure 1).

### Complex Decongestive Therapy

On the day of admission, patients were informed about the purpose and procedures for CDT. The short-term reduction phase of CDT consisted of multi-layered compression bandaging (MLCB), manual lymphatic drainage (MLD), remedial exercise, and skin care which started the day after hospitalization.

One physical therapist applied the MLCB to the affected limb and it was maintained for more than 22 h a day except during the MLD and to clean the limb. Each morning, one physiatrist unwrapped the MLCB, checked the skin condition and measured the circumference of the unaffected and affected limbs. Subsequently, the physical therapist performed morning MLD and immediately after MLCB was applied to the affected limb. Remedial exercise, including stretching, aerobic exercise, and resistance exercise were performed under the instruction of the therapist for 90 min per day. Prior to afternoon MLD, the therapist unwrapped MLCB again, and the skin condition was evaluated. After washing the body, the physical therapist applied MLCB which was kept until the next morning.

Nutrition management was also conducted in consultation with nutritionists. One physical therapist who specialized in lymphedema treatment performed MLD twice per day for 30 min. CDT was terminated when there was no change in the CM for 3 or more consecutive days.

### Lymphedema Evaluation

Lymphedema was evaluated via both BIA and CM at the initial admission (initial) and before discharge (follow-up, FU). For upper extremity measurements, the circumference was measured at both 10 cm above and below the elbow crease, and wrist while the patient was sitting with the elbow extended. For lower extremity measurements, the circumference was measured at both 10 cm, 20 cm above, and 10 cm below the lower border of the patella and ankle while the patient was supine with the knee extended. All measurements were performed three times by one physiatrist, and the average value was recorded. The sum of arm or leg circumference was calculated by summing the perimeters measured at the above-mentioned positions.

BIA was measured via a multi-frequency impedance plethysmograph body composition analyzer (Inbody720, Biospace, Seoul, Korea) by one experienced medical laboratory technician. Impedance was measured at a frequency of 1 kHz in both the affected and unaffected limb which reflect the lymph volumes. The ratio of extracellular water (ECW) to total body water (TBW) which represent status of ECF (extracellular fluid) and edema was obtained. It categorized as follows: constant state-0.38, mild overhydrated state-0.390–0.399, and moderate to severe overhydrated state ≥0.400) (19).

The inter-limb ratios (ILRs) of CM or impedance were calculated based on below formula at the beginning and the end of hospitalization. These initial and FU results of ILR were compared.

$$\begin{array}{l} Inter-limb\;ratio=\\ \;affected\;limb\;measurement÷unaffected\;limb\;measurement \end{array}$$

The limb volume was derived from the CM using the truncated cone formula. The relative percentage increase of volume (%RVI) of affected limb compared to unaffected limb was defined as [(affected arm or leg volume/unaffected arm or leg volume)−1]^*^100. Patients were classified by their initial %RVI as mild (<15%), moderate (15–36%), or severe (>37%) (20). According to lymphedema severity, the effects of CDT were compared. The cutoff values of ECW/TBW were obtained using receiver-operating characteristic (ROC) curve for detecting mild and severe degree of CTRL.

This study was approved and conducted in accordance with the recommendations of the institutional review board (IRB) of our university hospital (approval number: 2017-02-024). Informed consent was obtained from all participants prior to conducting any measurements.

### Statistical Analysis

Simple descriptive statistics and frequency analysis were used to characterize the samples and the distribution of variables. Data are presented as median (interquartile range) for continuous variables and all parameters of clinical outcome and BIA. The initial and FU circumference, and volume were compared by Wilcoxon signed-rank test. The absolute values of CM and volume between the affected and the unaffected side were also compared by Wilcoxon signed-rank test. For comparisons of changes in ILR of circumference, volume, and impedance among three severity groups during short-term in-hospital stay, one-way analysis of variance (ANOVA) test was used. Repeated measures ANOVA was used for both the intra-group and intergroup comparison of the CDT effects depending on lymphedema severity. For comparison of values among three groups at each time, one-way ANOVA and the Bonferroni *post-hoc* test were used.

The cutoff values were obtained using ROC curves for calculated ECW/TBW of mild and severe CTRL. Sensitivity and specificity were calculated with cutoff values of the BIA.

Spearman's correlation coefficients were calculated to investigate associations among parameters. Statistical analysis was performed using standard statistical software (SPSS version 21.1 for Windows, SPSS, Inc., Chicago, IL, USA). The significance level was set at 0.05 for all comparisons.

## Results

A total of 29 patients, 12 of whom had CTRL in an upper extremity and 17 of whom had CTRL in a lower extremity, were included in this analysis. All patients had undergone previous surgical treatment for breast or gynecological cancer. The median time from the onset of lymphedema to CDT was 8.0 months. The median duration of CDT was 9 days (9.0 days for mild, 11.5 days for moderate, and 19.5 days for severe degree of CTRL; Table 1).

**Table 1:** Characteristics of patients with lymphedema.

**Parameters**	
Age (years)	54.0 (47.5–61.0)
Treatment method	
Surgery	29 (100)
CTx	6 (20.7)
RTx	0
CTx+RTx	18 (62.1)
No CTx or RTx	5 (17.2)
Upper/Lower affected limb	12/17 (41.4/58.6)
Right/Left affected limb	13/16 (44.8/55.2)
Time to CDT (months)	8.0 (5.5–14.0)
Upper extremity	7.0 (2.8–13.3)
Lower extremity	8.0 (6.0–16.0)
Duration of CDT (days)	9.0 (9.0–15.0)
Mild (*n* = 13)	9.0 (9.0–15.0)
Moderate (*n* = 12)	11.5 (8.0–12.5)
Severe (*n* = 4)	19.5 (11.3–24.0)

*Values are reported as the median (interquartile range) or numbers (%). CTx, Chemotherapy; RTx, Radiotherapy; CDT, complex decongestive therapy; n, numbers*.

**Figure 2:**
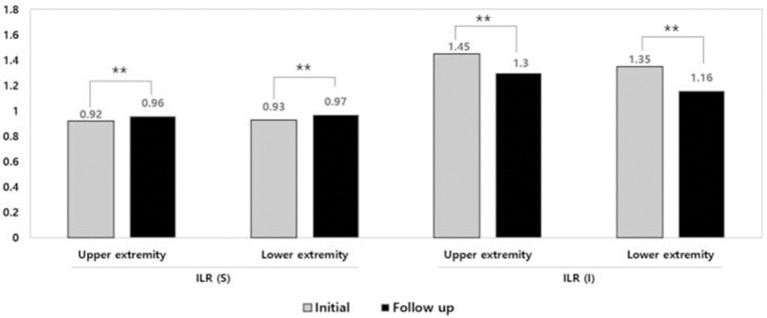
Improvement of inter-limb ratio of sum of circumference and impedance after complex decongestive therapy. **p < 0.01. ILR, inter-limb ratio; S, Sum of circumference; I, Impedance.

At initial assessment, the circumference and volume were significantly different between the affected and unaffected limb in both upper and lower extremities (Table 2, *p* < 0.05). There was a significant improvement in the absolute value of circumference, volume, and ECW/TBW and ILR of circumference, volume, impedance after CDT (Table 2, Figure 2, *p* < 0.01, *p* < 0.05).

**Table 2:** Change in evaluation parameters after complex decongestive therapy.

	**Initial**	**Follow up**	***P* value**
Lymphedema severity			
Mild/moderate/severe	12/13/4	22/7/0	
Weight (kg)	62.5 (58.3–67.8)	62.8 (56.8–68.5)	0.057
BMI (kg/m^2^)	25.0 (23.5–28.6)	25.7 (23.2–28.7)	0.016^*^
Body fat (%)	34.4 (28.8–40.2)	34.2 (28.9–40.9)	0.572
Body fat (kg)	23.1 (16.7–30.00)	22.7 (17.6–28.2)	0.909
ECW/TBW	0.39 (0.386–0.397)	0.39 (0.385–0.395)	0.009^**^
Sum of circumference (cm)			
Upper extremity			
Affected limb	90.1 (85.9–99.3)	87.0 (81.6–93.4)	0.002^**^
Unaffected limb	85.5 (81.3–87.4)	83.7 (80.1–86.5)	0.015^*^
*p* value	0.002^††^	0.002^††^	
Lower extremity			
Affected limb	158.0 (132.0–169.8)	152.5 (144.5–157.7)	0.005^**^
Unaffected limb	146.5 (123.5–153.8)	142.0 (138.5–150.8)	0.016^*^
*p* value	<0.001^††^	<0.001^††^	
Volume (L)			
Upper extremity			
Affected limb	11.4 (10.4–13.7)	10.8 (9.6–12.2)	0.002^**^
Unaffected limb	9.8 (9.1–10.8)	9.8 (9.0–10.5)	0.041^*^
*p* value	0.002^††^	0.002^††^	
Lower extremity			
Affected limb	49.1 (37.3–56.9)	44.8 (33.6–44.8)	<0.001^**^‘
Unaffected limb	40.8 (33.5–46.9)	39.0 (31.1–44.0)	0.001^**^
*p* value	<0.001^††^	<0.001^††^	
ILR (S)			
Upper extremity	0.92 (0.90–0.96)	0.96 (0.93–0.98)	0.003^**^
Lower extremity	0.93 (0.91–0.95)	0.97 (0.93–0.97)	0.001^**^
ILR (V)			
Upper extremity	0.84 (0.78–0. 89)	0.91 (0.85–0.93)	0.002^**^
Lower extremity	0.89 (0.82–0.91)	0.93 (0.87–0.97)	0.001^**^
ILR (I)			
Upper extremity	1.45 (1.11–1.58)	1.3 (1.08–1.45)	0.008^**^
Lower extremity	1.35 (1.16–1.56)	1.16 (1.10–1.29)	0.001^**^

*Values are reported as the median (interquartile range)*.

* *p < 0.05*,

** *p < 0.01, compared to the initial assessment*.

†† *p < 0.01, compared to the unaffected limb. BMI, body mass index; ILR, inter-limb ratio; S, Sum of circumference; V, Volume; I, Impedance; ECW, extracellular water; TBW, total body water*.

Initial absolute value of circumference of affected limb was significantly correlated with initial impedance in affected (*r* = −0.651^**^) and unaffected (*r* = −0.841^**^) limb, absolute value of circumference in unaffected limb (*r* = 0.953^**^) and follow up (FU) absolute value of circumference in affected (*r* = 0.971^**^) and unaffected (*r* = 0.944^**^) limb and impedance in affected (*r* = 0.593^**^) and unaffected (*r* = −0.778^**^). Initial ILR (I) was significantly correlated with initial ECW/TBW (*r* = 0.649^**^), absolute value of impedance of affected limb (*r* = −0.745^**^) and ILR (S) (*r* = −0.820) and FU ECW/TBW (*r* = 0.591^**^), absolute value of impedance of affected (*r* = −0.766^**^) and unaffected (*r* = −0.387^*^) limb and ILR (S) (*r* = −0.778) and ILR (I) (*r* = 0.896^**^) (Table 3). It is consistent with the findings of previous studies (15, 21).

**Table 3:** Correlations between initial and follow-up inter-limb ratios of circumference, impedance, and extracellular fluid status.

	**Initial**			**Follow up**		
	**ILR (S)**	**ILR (I)**	**ECW/TBW**	**ILR (S)**	**ILR (I)**	**ECW/TBW**
**INITIAL**						
ILR (S)	1	−0.820^**^	−0.622^**^	0.826^**^	−0.833^**^	−0.529^**^
ILR (I)	−0.820^**^	1	0.649^**^	−0.778^**^	0.896^**^	0.591^**^
ECW/TBW	−0.622^**^	0.649^**^	1	−0.508^**^	0.512^**^	0.798^**^
**FOLLOW-UP**						
IRL (S)	0.826^**^	−0.778^**^	−0.508^**^	1	−0.816^**^	−0.461^*^
IRL (I)	−0.833^**^	0.896^**^	0.512^**^	−0.816^**^	1	0.499^**^
ECW/TBW	−0.529^**^	0.591^**^	0.798^**^	−0.461^*^	0.499^**^	1

* *p < 0.05*,

** *p < 0.01 by Spearman's correlation. ILR, inter-limb ratio (unaffected side/affected side); S, Sum of circumference; I, Impedance; ECW, extracellular water; TBW, total body water*.

Initial and FU ECW/TBW and ILRs for circumference, and impedance revealed significant strong correlations with each other (Table 3, *r* > 0.7, *p* < 0.01).

When patients were divided according to the severity of their CTRL volume, nine patients (75.0%) among 12 patients with initially moderate degree of CTRL improved to mild degree after CDT. Similarly, all four patients originally classified as severe degree of CTRL were improved to moderate degree of CTRL after CDT. Finally, 22 patients were classified as mild degree and 7 patients as moderate degree after CDT.

**Figure 3:**
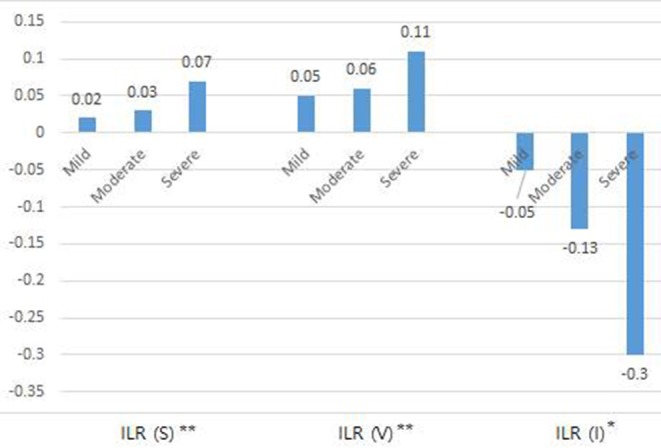
Change of inter-limb ratio of sum of circumference, volume, impedance between initial and follow up evaluation after complex decongestive therapy according to the severity of lymphedema. *p < 0.05, **p < 0.01. ILR, inter-limb ratio; S, Sum of circumference; V, Volume; I, Impedance.

The initial values of relative percentage increase in sum of circumference (%RSI), volume (%RVI), impedance (%RII), inter-limb ratios of circumference, volume, and impedance (*p* < 0.001^**^) and ECW/TBW (*p* = 0.004^**^) revealed significant inter-group difference among three severity groups. Also, significant improvements in the %RSI, %RVI, %RII, ECW/TBW, and absolute values of ILRs of circumference and impedance were induced by CDT (Table 4). Changes in ILR of sum of circumference (*p* < 0.001^**^), volume (*p* = 0.002^**^), and impedance (*p* = 0.046^*^) between initial and FU evaluation after CDT according to the severity of lymphedema was significantly different among three groups according to severity (Figure 3).

**Table 4:** Changes of parameters of circumference, volume, impedance, and extracellular fluid status after complex decongestive therapy according to the severity of lymphedema.

	**Initial**	**Follow up**	**Time effect *p* value**	Time^*^group effect ***p*** **value**	
%RSI			<0.001^**^	<0.001^**^	
Mild (*n* = 13)	4.9 (2.6–6.1)	2.4 (1.6–3.2)			<0.001^§§^
Moderate (*n* = 12)	9.2 (7.9–10.1)	4.9 (3.6–6.5)		<0.001^††^	
Severe (*n* = 4)	19.6 (17.4–21.0)	10.4 (9.4–11.2)		<0.001^††^	<0.001^§§^
%RVI			<0.001^**^	<0.001^**^	
Mild (*n* = 13)	10.7 (7.1–12.1)	4.4 (2.5–6.9)			<0.001^§§^
Moderate (*n* = 12)	20.0 (18.8–24.8)	12.1 (9.5–16.5)		<0.001^††^	
Severe (*n* = 4)	45.8 (40.2–68.1)	28.0 (23.2–32.1)		<0.001^††^	<0.001^§§^
%RII			<0.001^**^	0.05	
Mild (*n* = 13)	11.4 (7.3–33.7)	8.6 (6.4–16.3)			0.003^§§^
Moderate (*n* = 12)	44.7 (32.7–58.0)	29.9 (15.4–44.2)		0.003^††^	
Severe (*n* = 4)	81.6 (59.2–104.3)	64.4 (39.7–66.3)		<0.001^††^	0.019^§^
ECW/TBW			0.043^*^	0.364	
Mild (*n* = 13)	0.39 (0.38–0.39)	0.39 (0.38–0.39)			0.177
Moderate (*n* = 12)	0.40 (0.39–0.40)	0.39 (0.38–0.40)		0.177	
Severe (*n* = 4)	0.41 (0.39–0.42)	0.40 (0.39–0.41)		0.007^††^	0.167
ILR (S)			<0.001^**^	<0.001^**^	
Mild (*n* = 13)	0.95 (0.94–0.97)	0.98 (0.97–0.98)			<0.001^§§^
Moderate (*n* = 12)	0.91 (0.91–0.93)	0.95 (0.93–0.97)		<0.001^††^	
Severe (*n* = 4)	0.84 (0.83–0.85)	0.91 (0.90–0.92)		<0.001^††^	<0.001^§§^
ILR (V)			<0.001^**^	<0.001^**^	
Mild (*n* = 13)	0.90 (0.89–0.93)	0.96 (0.93–0.98)			<0.001^§§^
Moderate (*n* = 12)	0.83 (0.80–0.84)	0.89 (0.86–0.91)		<0.001^††^	
Severe (*n* = 4)	0.69 (0.60–0.71)	0.78 (0.75–0.81)		<0.001^††^	<0.001^§§^
ILR (I)			<0.001^**^	0.046^*^	
Mild (*n* = 13)	1.1 (1.08–1.34)	1.09 (1.07–1.16)			0.003^§§^
Moderate (*n* = 12)	1.45 (1.33–1.58)	1.30 (1.16–1.45)		0.003^††^	
Severe (*n* = 4)	1.82 (1.59–2.04)	1.64 (1.39–1.66)		<0.001^††^	0.021^§^

*Values are reported as the median (interquartile range)*.

* *p < 0.05*,

** *p < 0.01, compared to patients among three groups*.

†† *p < 0.01, compared to patients with mild lymphedema by the Bonferroni post-hoc test*.

§ *p < 0.05*,

§§ *p < 0.01, compared to patients with moderate lymphedema the Bonferroni post-hoc test. %RSI, relative percentage sum of circumference increase %RVI, relative percentage volume increase; %RII, relative percentage impedance increase; ILR, inter-limb ratio; S, Sum of circumference; I, Impedance*.

Initial and FU ECW/TBW showed moderate to high correlation with initial and FU %RSI, %RVI, and %RII (Table 5).

**Table 5:** Correlations of initial and follow-up ECW/TBW with a relative percentage increase of circumference, volume, and impedance.

	**Initial**				**Follow up**			
	**ECW/TBW**	**%RSI**	**%RVI**	**%RII**	**ECW/TBW**	**%RSI**	**%RVI**	**%RII**
**INITIAL**								
ECW/TBW	1	0.633^**^	0.580^**^	0.650^**^	0.798^**^	0.534^**^	0.526^**^	0.529^**^
%RSI	0.633^**^	1	0.902^**^	0.835^**^	0.522^**^	0.841^**^	0.753^**^	0.856^**^
%RVI	0.580^**^	0.902^**^	1	0.783^**^	0.492^**^	0.770^**^	0.867^**^	0.768^**^
%RII	0.650^**^	0.835^**^	0.783^**^	1	0.590^**^	0.766^**^	0.705^**^	0.898^**^
**FOLLOW UP**								
ECW/TBW	0.798^**^	0.522^**^	0.492^**^	0.590^**^	1	0.465^**^	0.522^**^	0.508^**^
%RSI	0.534^**^	0.841^**^	0.770^**^	0.766^**^	0.465^*^	1	0.845^**^	0.791^**^
%RVI	0.526^**^	0.753^**^	0.867^**^	0.705^**^	0.522^**^	0.845^**^	1	0.699^**^
%RII	0.529^**^	0.856^**^	0.768^**^	0.898^**^	0.508^**^	0.791^**^	0.699^**^	1

* *p < 0.05*,

** *p < 0.01 by Spearman's correlation*.

*ECW, extracellular water; TBW, total body water; %RSI, relative percentage increase of sum of circumference; %RVI, relative percentage increase of volume; %RII, relative percentage increase of impedance*.

The cutoff value for ECW/TBW which distinguish mild degree of CTRL from other degrees of CTRL was 0.3885. The area under the curve (AUC) was 0.800 (*p* = 0.006^**^) and the sensitivity and specificity were 87.5 and 69.2%, respectively. The cutoff value for ECW/TBW which distinguish severe degree of CTRL from other degrees of CTRL was 0.3955. The area under the curve (AUC) was 0.820 (*p* = 0.043^*^) and the sensitivity and specificity were 75.0 and 76.0%, respectively.

## Discussion

Lymphedema is composed of accumulated protein, fluid, fibrotic, and fatty tissues in the extravascular interstitium which is influenced by stage of CTRL. BIA can differentiate ECF from total limb volume and is useful for early diagnosis and assessing severity of lymphedema (17, 22–25).

Body composition and ECF status should be carefully considered for determining the CDT effects on CTRL. However, to the best of our knowledge, there are few studies to report the feasibility of BIA as a tool of serial evaluation for treatment outcomes in patients with lymphedema.

In this study, CDT induced significant improvement of absolute value or ILR of circumference, calculated volume, impedance, and ECW/TBW in CTRL (Tables 2, 4). In addition, we identified a strong relationship between the ILR of impedance of BIA, the ILRs of circumference and ECF status at the initial and FU evaluation, respectively (Table 3). Furthermore, initial and FU ECF status and a relative percentage increase of circumference, volume, and impedance significantly correlated with each other (Table 5). Therefore, it suggests that BIA may be a useful tool to monitor not only changes in circumference, but also changes in body composition in CTRL therapy.

When patients were grouped by severity, there were significant differences in ECW/TBW, %RSI, %RVI, %RII, ILR of circumference, volume, and impedance according to severity at initial assessment. Also, there were significant intergroup differences according to time, except for ECW/TBW. ECW/TBW were significantly improved in CTRL, regardless of severity (Table 4). Furthermore, 75.0% of patients classified with moderate CTRL improved to mild CTRL after CDT, and 100% of patients originally classified with severe CTRL improved to moderate CTRL after CDT. Although patients with severe CTRL underwent a longer duration of treatment (by ~1 week) than patients with mild or moderate CTRL (Table 1), it suggests that patients with severe lymphedema might expect favorable outcomes with short-term intensive CDT.

Interestingly, parameters in unaffected limb as well as affected limb showed changes after CDT. As high pre-treatment BMI (>30) is a well-established risk factor for lymphedema (26, 27), our CDT program included weight management during CDT. However, there were no significant changes of weight or body fat contents after CDT (Table 2), despite BMI is changed significantly at FU (Table 2). Because the number of patients participated in the current study was relatively small and the BMI of all patients ranged <30 and the duration of treatment was as short as 12 days, the effect of our program on weight management remains uncertain to draw conclusions. Thus, the improvement of circumference, volume, and impedance of the unaffected limb as well as the affected limb at FU is thought to be related to the improvement of edema status which is represented by ECW/TBW (Tables 2, 5). Further research should be needed to determine whether reducing BMI affects treatment outcomes, especially because the management of lymphedema is a lifelong issue.

Previous studies have focused primarily on BIA as a diagnostic tool for lymphedema, with several studies highlighting its utility for early diagnosis of lymphedema (17, 22, 23). One study reported that BIA was associated with nearly 100% sensitivity and specificity for early detection of lymphedema (17). In contrast, another study reported the sensitivity and specificity of bioimpedance compared to volume displacement as 75 and 93%, respectively (28). These studies indicate that clinical and laboratory methods are needed in addition to BIA to accurately diagnosis lymphedema. While these studies focused on upper limb lymphedema, several studies on gynecological cancer employed BIA for lower limb lymphedema (29). However, the utility of BIA in these studies were to establish an early detection of lymphedema, perioperative fluid imbalance, or postoperative complications. Therefore, additional studies are needed to test the sensitivity and specificity of BIA for evaluation of CDT effect on lower extremity lymphedema.

Notably, the cutoff value of ECW/TBW were calculated as 0.3885 (moderate) and 0.3955 (severe) which were slightly lower, compared with the category of simple edema (19). It might be resulted from the composition of lymphedema contents. Lymphedema is a chronic and progressive condition that requires constant management over the lifetime (30, 31). Patients with lymphedema who have completed the short-term reduction phase of CDT enter the long-term maintenance phase of CDT. Our results demonstrated the possibility of BIA parameters as monitoring indicator in both upper and lower limbs lymphedema. The outcome from the short-term reduction phase becomes the patient's new baseline. When a change from this baseline occurs during the long-term maintenance phase, then the patient must return to the reduction phase of CDT. It is important to detect small changes as soon as possible, because small changes require shorter durations of treatment, and early detection of minute changes in lymphedema are associated with more favorable long-term outcomes and improved quality of life and function (9, 10, 32). Circumference and volume measurement are commonly used to monitor lymphedema during the maintenance phase because they are easy and cost effective to conduct. But volume measurement needs specific equipment such as perometer or water displacement volumetry. BIA also is a non-invasive measure and is convenient for both the physician and the patients. Reference values for healthy populations and standards for diagnosis of lymphedema have been established for BIA (33). However, considering individual variability especially noted for obese individuals (34), the patient's longitudinal value after CDT can be used as a reference during long-term maintenance.

This study has several limitations. A relatively small number of patients were included in the analysis. One advantage, however, is that our cohort included patients with lymphedema in both the upper and lower extremities. In this study, we assessed BIA in the evaluation of improvements before and after the reduction phase of CDT. Long-term studies should evaluate whether BIA is a reliable tool of continuous monitoring during the maintenance phase of CDT. The number of circumference measurements performed on each limb is too few to calculate accurate volume using truncated cone formula. While this study primarily focused on trends in segmental volume change, the data in BIA reflects whole arm or leg. Therefore, future study is desirable to measure the circumference of more areas or use segmental perometry for accurate volume estimation. In addition, due to small numbers of participants, we couldn't perform subgroup analysis according to lymphedema stages. Because BIA results can vary depending on the condition of the skin, the clinical value of BIA might be emphasized on the higher stages. Thus, further research is needed to understand whether the interpretation of BIA values depend on not only the degree of lymphedema but also the stages of lymphedema, especially among patients with terminal stage of lymphedema with irreversible skin changes.

In conclusion, BIA is practical to measure lymphedema treatment outcomes. BIA is more sensitive for detecting subtle changes after CDT than CM or volume measurement in relation to ECF status. Therefore, it would be useful for the long-term maintenance care of lymphedema as well. Despite our small sample size, estimated improvement in BIA of patients with mild, moderate, and severe lymphedema was ~0.02, 0.03, and 0.07 in inter-limb ratio of circumference sum and −0.05, −0.13, −0.3 in inter-limb ratio of impedance, respectively, after treatment. Based on the results of this study, it is necessary to determine whether BIA can be used for long-term monitoring of lymphedema, which would facilitate long-term management of this condition and ultimately improve the quality of life of patients with lymphedema.

## Data Availability Statement

The datasets generated for this study are available on request to the corresponding author.

## Ethics Statement

The studies involving human participants were reviewed and approved by IRB of CHA Bundang Medical Center (approval number: 2017-02-024). The patients/participants provided their written informed consent to participate in this study.

## Author Contributions

KC and EH: conception and design, collection and/or assembly of data, data analysis and interpretation, and manuscript writing. SL, HP, and CL: provision of study material or patients, data analysis, and interpretation. SI: conception and design, data analysis and interpretation, manuscript writing, and final approval of manuscript.

### Conflict of Interest

The authors declare that the research was conducted in the absence of any commercial or financial relationships that could be construed as a potential conflict of interest.
